# Associations Between Brominated Flame Retardant Exposure and Depression in Adults: A Cross-Sectional Study

**DOI:** 10.3390/toxics12120918

**Published:** 2024-12-19

**Authors:** Yulan Cheng, Yue Fei, Zemin Xu, Ruiyao Huang, Yuling Jiang, Lihan Sun, Xuehai Wang, Shali Yu, Yonghua Luo, Xiaobo Mao, Xinyuan Zhao

**Affiliations:** 1 Affiliated Haimen Hospital of Xinglin College, Nantong University, Nantong 226019, China; nanyu295@163.com; 2 Nantong Key Laboratory of Environmental Toxicology, Department of Occupational Medicine and Environmental Toxicology, School of Public Health, Nantong University, Nantong 226019, China; 2317320024@stmail.ntu.edu.cn (Y.F.); 2317320014@stmail.ntu.edu.cn (Z.X.); 18901533561@163.com (Y.J.); lankeqing3910@gmail.com (X.W.); 7693539@ntu.edu.cn (S.Y.); 3 Department of Clinical Medicine, Nantong University Xinglin College, Nantong 226000, China; hry20041014@163.com; 4 School of Medicine, Nantong University, Nantong 226001, China; sunlihan2024@163.com; 5 Nantong Fourth People’s Hospital, Nantong 226019, China; ntluoyonghua@163.com

**Keywords:** NHANES, brominated flame retardants, depression, chemical mixture exposure, quantile-based g computation

## Abstract

Background: Brominated flame retardants (BFRs) are a type of widespread pollutant that can be transmitted through particulate matter, such as dust in the air, and have been associated with various adverse health effects, such as diabetes, metabolic syndrome, and cardiovascular disease. However, there is limited research on the link between exposure to mixtures of BFRs and depression in the general population. Methods: To analyze the association between exposure to BFRs and depression in the population, nationally representative data from the National Health and Nutrition Examination Survey (NHANES; 2005–2016) were used. In the final analysis, a total of 8138 adults aged 20 years and older were included. To investigate the potential relationship between BFRs and outcomes, we used binary logistic regression, restricted cubic spline (RCS), quantile-based g computation (QGC), and weighted quantile sum (WQS) regression. Results: The findings showed that serum BFR concentrations were associated with depressive symptoms over a broad spectrum. Binary logistic regression and RCS analysis showed that certain BFRs, particularly PBB153, were significantly and positively associated with the incidence of depression, even after adjustment for various confounders (*p* < 0.05). Mixed exposure to BFRs was also found to be associated with depression in the population, with a stronger association in men. The two most influential BFRs, PBB153 and PBDE85, were identified in both mixed exposure models and are potential risk factors of concern. Conclusion: Our study identified new insights into the relationship between BFRs and depression, but sizable population-based cohort studies and toxicology mechanism studies will be needed to establish causality.

## 1. Introduction

Brominated flame retardants (BFRs) are a class of material additives widely used in various consumer products, including children’s toys, electronics (such as mobile phones, computers, and television cords), automobiles, kitchen appliances, apparel, carpets, rugs, cushions, upholstery, and textiles, as well as in housing, building materials, and a wide range of plastics, which are added to reduce the likelihood of flammable materials catching fire [1,2]. Research has indicated that certain BFRs are slowly released into the air over time due to their semi-volatile nature or product abrasion, accumulating in dust through gas-dust partitioning [3]. Intake of BFRs through airborne particles, such as dust, has been identified as a critical route of exposure for humans, posing a particular risk to the health of children and occupational workers [2,4]. The estimated average (median) exposure to total BFRs from dust intake was 2.8 ng/kg bw/day (1.3 ng/kg bw/day) for UK adults and 61 ng/kg bw/day (30 ng/kg bw/day) for UK toddlers [5]. Additionally, HBCD (Hexabromocyclododecane) concentrations in breast milk from Swedish women ranged between 0.086 and 0.36 ng/g lw [6]. Meanwhile, based on extensive data on FR (Flame Retardant) levels from the USA and China, modeling assessments show that the total median daily absorbed dose (DAD) value of BFRs is significantly higher in the USA than in China [7]. In fact, products containing BFRs are released into various environments through emissions, high-temperature releases, and the transformation of photodegradation products, and these BFRs have even been detected in places far from their production or use, as well as in humans and other organisms [8]. Meanwhile, growing concerns regarding the persistence, bioaccumulation, and potential toxic effects of BFRs on the environment and human health have prompted several international organizations and European countries to prohibit some of these substances under the precautionary principle [9]. Four BFRs have been listed as POPs for worldwide restrictions under the United Nations Environment Programme (UNEP) Stockholm Convention since the beginning of this century: tetra/penta (Penta-mix) and hexa/hepta (Octa-mix) related brominated diphenylethers (PBDEs), decabromodiphenylether (DecaBDE), and hexabromocyclododecane (HBCD or HBCDD) [10]. Evidence from experimental studies and population data suggests that BFRs have been linked to a variety of adverse health effects, including reproductive health problems, immune system dysfunction, diabetes mellitus, metabolic syndrome, thyroid disorders, and cardiovascular disease [6,11,12]. There is also some evidence that exposure to BFRs can also have adverse neurological effects. For instance, Chen et al. found that exposure to tetrabromobisphenol A (TBBPA) in zebrafish during the developmental window may disrupt various molecular pathways and lead to neurobehavioural deficits [13]. The neurotoxicity and amyloidogenicity of BFRs in vitro have also been demonstrated by Fawaz et al. using BFR exposure experiments on SH-SY5Y neuronal cells [14]. The main sources of BFR exposure for the general adult public were reported as room dust and consumption of fish and dairy foods, while breast milk is the main route of exposure for children, and recycling of electronic waste, etc., is an important route of exposure to BFRs for occupational personnel [15,16]. The vast majority of BFRs have low water solubility and high lipophilicity, and their high lipophilicity may lead to significant deposition in organs rich in adipocytes after exposure [17]. At the same time, the brain has a high lipidity, high oxygen consumption, and low antioxidant capacity, making it particularly susceptible to the uptake of lipophilic toxicants into the brain tissue [18,19].

Depression is a common mental illness caused by a complex interaction of social, psychological, and biological factors that can affect anyone and is characterized by long periods of low mood or loss of pleasure or interest in activities [20]. Often, individuals with depression frequently exhibit disrupted sleep patterns, diminished appetite, low self-esteem, contemplations of mortality, and pessimism about the future. In 2008, the World Health Organization (WHO) classified major depressive disorder (MDD) as the third-leading cause of the global disease burden, with the prediction that by 2030 it would become the leading cause [21]. Epidemiological studies have indicated that major depression is a prevalent phenomenon, with approximately one in five individuals exhibiting an episode during their lifetime [22]. In 2022, the annual prevalence of major depression among US adults was approximately 7.0% among men and 10.4% among women [23]. In terms of gender, females experience higher rates of dysphoria, anxiety disorders, and major depression than males from adolescence onwards [24]. Previous studies have shown that depression may develop due to various factors, including poor upbringing during childhood, negative stress coping during adolescence, psychosocial adversity during old age, and perinatal depression, which is specific to women [25,26,27,28]. Corresponding studies have also shown that environmental pollutants are neurotoxic and are associated with depression and structural brain dysfunction [29]. Yi and colleagues observed a positive correlation between serum concentrations of perfluoroalkyl and poly-fluoroalkyl substances (PFAS) and elevated scores on the Patient Health Questionnaire-9 (PHQ-9), a depression screening tool [30]. Research has also demonstrated that exposure to PAHs is linked to depression in females who are going through perimenopause [31]. However, currently, there is no evidence linking exposure to BFRs with an increased risk of depression in the population.

Therefore, this study sought to investigate the association between exposure to BFRs, as indicated in data from the 2005–2016 National Health and Nutrition Examination Survey (NHANES), and the risk of developing depression. We used logistic regression and restricted cubic spline (RCS) to analyze linear and nonlinear associations between a single BFR and outcome, respectively. Also, to explore the combined effect of BFR mixtures on outcomes and the importance ranking of each BFR, we used quantile-based g computation (QGC) and weighted quantile sum (WQS) regression models, as BFRs were present in the mixtures.

## 2. Methods

### 2.1. Study Population

**Figure 1:**
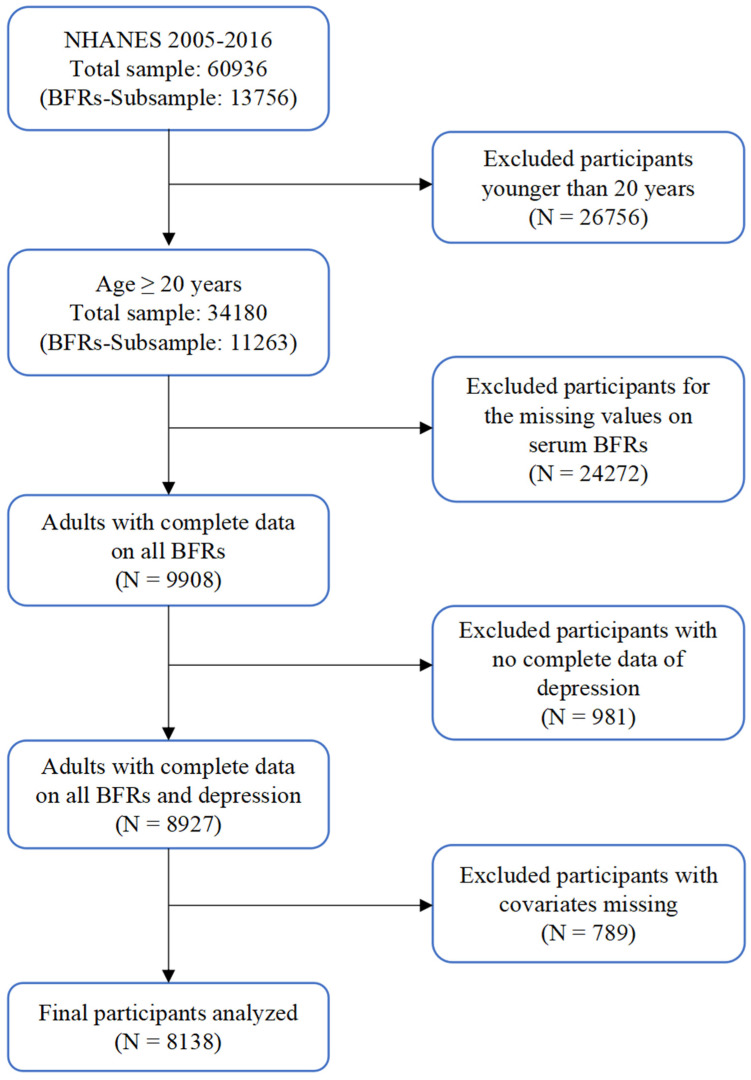
Flow diagram for selecting eligible participants from NHANES.

Since 1999, the NHANES has investigated the health and nutritional status of the general US population every 2 years. These data are used to determine the population prevalence of specific diseases (acute and chronic), nutritional intake and status, and chemical exposure. Important information can be obtained with a sample size of around 10,000 people per cycle, including home interviews, standardized physical examinations, medical history, and biological specimens, some of which are used to measure exposure to environmental contaminants. The random sample maintained the survey’s representative design based on the complex principle of stratified multistage sampling. The study protocol was approved by the National Center for Health Statistics Institutional Review Board, and all survey participants provided informed, written consent. Initially, we collected information on 60,936 participants from the NHANES 2005–2016 cycle, including all required variables. The study content established certain inclusion and exclusion criteria to screen participants, which included their age, serum BFRs, depression, and covariates. Finally, the analysis included 8138 adults aged 20 years and older (Figure 1). Given the significant number of participants excluded due to age criteria and absent BFRs sample data, we gathered a total of 25,535 participants across 6 time periods, all of whom had complete data on baseline characteristics, various covariates, and depression assessments for statistical analysis. Among these, 8138 participants also had complete serum BFR data (i.e., the population we included in our study), while the remaining 17,397 did not possess complete serum BFR data. The results indicated that both populations were generally aligned across various variable levels, and the 8138 participants in our study well represented the relationship between BFR exposure and depression among US adults (Table S1).

### 2.2. Depression Definition

NHANES uses a patient health questionnaire to assess participants’ depressive mood severity in the past 2 weeks. The questionnaire comprises nine questions, with each answer scored from zero to three to indicate the severity of symptoms, ranging from none to several per day. If a patient scores 10 or more points on the PHQ-9 questionnaire, it can be assumed that the study participant is experiencing depression [32]. Previous studies have shown that PHQ-9 sensitivity and specificity were 0.86 and 0.85 [33]. It has been extensively utilized in numerous cross-sectional studies to assess depression and has demonstrated good reliability and validity [30,34].

### 2.3. Measurement of Serum BFRs

The NHANES dataset offers information on PBB-153 and eleven kinds of serum polybrominated diphenyl ethers (PBDEs). The MEC Laboratory Procedures Manual provides detailed information on the quality control of sample data to ensure the availability and authenticity of these sample data [35]. Additionally, lipid-conditioned BFR data is available for researchers. Further details on the measuring instruments, data collection process, and data types can be found at the NHANES website [36]. To ensure the robustness and representativeness of the results, this study only included brominated flame retardants with a detection rate of over 75%. These include PBDE 28, PBDE 47, PBDE 85, PBDE 99, PBDE 100, PBDE 153, PBDE 154, PBDE 209, and PBB 153. Serum BFR concentrations below the lower limit of detection (LLOD) were estimated as the square root of half the LLOD value. The NHANES used a pool strategy in which serum samples from eight participants were collected in a pool for measurement of serum BFR concentrations. The statistical methodology employed in forming pools in this study ensures that the resulting measurements are mathematically identical to the weighted arithmetic mean of the pool samples. This follows the NHANES weighted sampling design, guaranteeing the accuracy of estimates representative of the overall US population [37].

### 2.4. Covariates

In addition to BFR compounds in serum, covariates were added to the analysis to account for demographic and behavioral differences among participants. Covariates, such as demographic characteristics (gender, age, race, educational level, marital status, and poverty income ratio (PIR)), lifestyle (BMI, cotinine level, and alcohol consumption), and questionnaire findings (self-reported hypertension and diabetes), were gathered via standardized questionnaires, physical and laboratory tests, and interviews with trained medical specialists. The measurement of cotinine concentrations provides a more precise indication of active or passive smoking status compared to the use of smoking questionnaires, thus offering a more accurate estimation of exposure to environmental tobacco smoke [12,38]. Serum cotinine concentrations below the lower limit of detection (LLOD) were calculated as the LLOD value divided by the square root of 2 [39].

### 2.5. Statistical Analyses

We performed descriptive analyses to determine the statistical measures of the data, such as the mean, standard deviation or median, and inter-quantile range (IQR) for continuous variables and the frequency distributions for categorical variables. We compared the baseline characteristics between groups based on depression status using the Chi-square tests for categorical variables and Student’s *t*-tests for continuous variables. Geometric means and medians were used to describe all participants’ serum concentrations of BFRs. Since serum BFR concentrations did not satisfy a normal distribution, we replaced the original concentrations with their ln-transformed values to better adapt to various statistical analysis models. Pearson correlation coefficients were also calculated between each pair of ln-transformed concentrations for the nine BFR chemicals. Moreover, to understand the trend of BFRs concentration in humans, the nine serum BFRs were expressed as geometric means and geometric standard deviations over the six data periods. The Mann–Kendall test was utilized to examine trends throughout the 12 years.

Weighted models were used in the multiple regression analyses, utilizing survey weights from the mobile exam center visit due to the complex, multistage sampling design used in NHANES. We divided participants into four groups based on quartiles of BFR concentrations, with the lowest quartile (Q1) being the reference group. ORs and 95% odds ratios (CIs) were calculated for the prevalence of depression in quartile 2 (Q2), quartile 3 (Q3), and quartile 4 (Q4) respondents compared to quartile 1 (Q1) for serum BFRs. We assigned the values of the BFRs for each of the four concentration intervals (Q1 to Q4) as the group median for their respective intervals (we used the median), and then performed a test for trend, with the resulting *p*-value reflecting whether there was a linear trend between the exposure and the outcome. Moreover, RCS curves with four knots (set at the 5th, 35th, 65th, and 95th percentiles of the BFRs metabolite concentrations) were plotted to examine the nonlinear relationship between levels of BFRs metabolites and depression. *P* for nonlinear values was calculated using the ‘anova’ function in the ‘rms’ package to assess the statistical significance of dose-response associations. QGC is built up upon WQS regression interpreting with g computation, which can be reliably used to investigate the joint effect of a high-dimensional mixture on a single outcome [40]. The quantiles of the exposure were set at 10, and the estimates obtained from models represented the effect of a decile increase in exposure mixtures. Finally, trends in the effect of this mixed exposure on outcomes were visualized using the QGC model.

### 2.6. Sensitivity Analysis

The factors leading to the occurrence of depression are complex and diverse, and gender differences in depression have been confirmed in several studies [41,42]. Therefore, we performed all regression analyses separately for men and women in the study to explore the potential effect of sex on the association between exposure and outcome. At the same time, the WQS model was utilized to examine and validate the relevant BFR ranks. WQS, like the QGC model, may be used consistently to investigate the combined impacts of high-dimensional mixtures on a single result. WQS assumes that the included exposures are linear and additive, with a one-way influence on the goal result [40]. We split the data into training (40%) and validation (60%). In the training dataset, we calculated the weight of each BFR using 1000 samples, and in the validation dataset, we evaluated it for mixed significance. In addition, since organohalogen compounds, including BFRs, are highly lipophilic, lipid-adjusted BFRs from NHANES were also used for all analyses. The lipid-corrected concentration was used to replace the initially determined BFR concentration to reduce the error introduced by the differences in lipid content between individuals [43].

We have included factors that may affect the results based on previous research and theoretical considerations [12,31]. In all analysis models, various potential confounders were adjusted (adjusted for gender, age, race, educational levels, marital status, PIR, BMI categories, cotinine levels, alcohol consumption, hypertension, and diabetes conditions). Analyses were conducted with R (4.2.2, https://cran.r-project.org/bin/windows/base/, accessed on 3 December 2024), and the QGC regression and RCS models were performed by R package “qgcomp” (version 2.10.1, https://CRAN.R-project.org/package=qgcomp, accessed on 3 December 2024) and “rms” (version 6.6.0, https://cran.r-project.org/web/packages/rms/index.html, accessed on 3 December 2024), respectively [44]. For all statistical analyses, bilateral *p*-values less than 0.05 were considered statistically significant.

## 3. Results

### 3.1. Basic Characteristics of Participants

Table 1 summarizes the fundamental features of the 8138 participants. A total of 716 participants developed depression; the frequency in women was 11.5%, which was about twice as high as in men (6.0%). Depressed individuals were more likely to be 40–60 years old, less educated, live alone, have a PIR ≤ 1.3, be obese, have hypertension, and have diabetes than non-depressed individuals.

Table 2 displays the geometric mean and quartile for serum BFRs. PBDE47 had the highest concentration of the nine serum BFRs, followed by PBDE153, and PBDE154 had the lowest concentration. Table S2 shows the difference in serum BFR concentration between different genders. We found that the serum BFR concentration in males was higher than that in females. Table S3 provided the detection rates for nine serum BFRs in each NHANES cycle. Figure S1 depicted the correlation between nine BFRs, with PBDE47 and PBDE99 showing the highest relevancy (r = 0.94). Table S4 shows the average BFR concentrations in the blood for each data release cycle. During the data release period, serum BFRs decreased significantly during 12 years (the majority of *p* for trend < 0.05).

### 3.2. Associations of Individual BFRs with Depression

**Figure 2:**
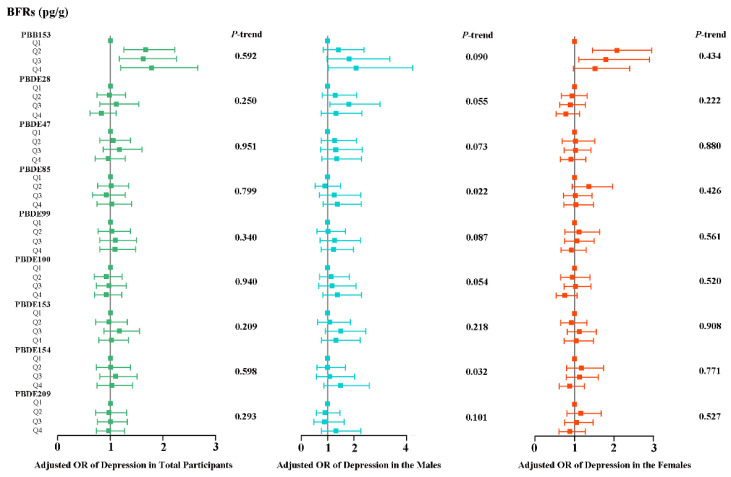
Weighted odds ratios (95% CIs) of depression by quartiles of ln-transformed serum BFR levels in all participants. The squares and horizontal lines represent the ORs and 95% CIs, respectively. All of the models are adjusted for demographic characteristics (age, race, educational levels, marital status, and PIR), lifestyle (BMI categories, cotinine levels, and alcohol consumption), and self-reported hypertension and diabetes conditions.

Figure 2 shows the results of logistic regression analyses of the link between nine single serum BFR concentrations and the prevalence of depression among adults. After adjusting a suite of confounding factors, some BFRs (especially PBB153) still showed a remarkable positive link with the prevalence of depression (*p* < 0.05). In detail, compared with Q1 of PBB153, the risk of depression all augmented, and the weighted OR with 95% CIs were 1.670 (1.254, 2.223), 1.627 (1.171, 2.259), and 1.787 (1.200, 2.661) for Q2, Q3, and Q4 respectively. For male participants, the Q4 of PBB153 was 2.086-fold odds with depression than Q1 (OR = 2.086; 95%CI: 1.026, 4.242). For female participants, compared with Q1 of PBB153, the risk of depression went up, and the weighted OR with 95% CIs were 2.076 (1.455, 2.960) and 1.797 (1.112, 2.905) for Q2 and Q3, respectively. Second, in male participants, PBDE28 was also discovered to be positively associated with depression risk at Q3 concentrations, with a weighted OR value of 1.798 (1.082, 2.988). However, no significant association was found between the remaining seven BFRs and the risk of depression.

Figures S2–S4 showed the results of the RCS analysis, suggesting a nonlinear association between partial ln converted serum BFR levels and depression risk. For instance, in the general population (Figure S2), PBDE28 showed a clear inverse U-shaped relationship with depression (*p* for nonlinear = 0.012). This was consistent with the trend in OR values of PBDE28 at different concentrations in Figure 2; that is, lower concentrations of PBDE28 were more positively associated with depression than higher concentrations. Meanwhile, in female participants (Figure S4), we found that at low concentrations of PBB153, women’s OR values for depression increased significantly (*p* < 0.05), but at high concentrations, this association became minor. Therefore, the link between low exposure to PBB153 and depression in women was noteworthy.

### 3.3. Associations of All BFRs with Depression in QGC Analyses

**Figure 3:**
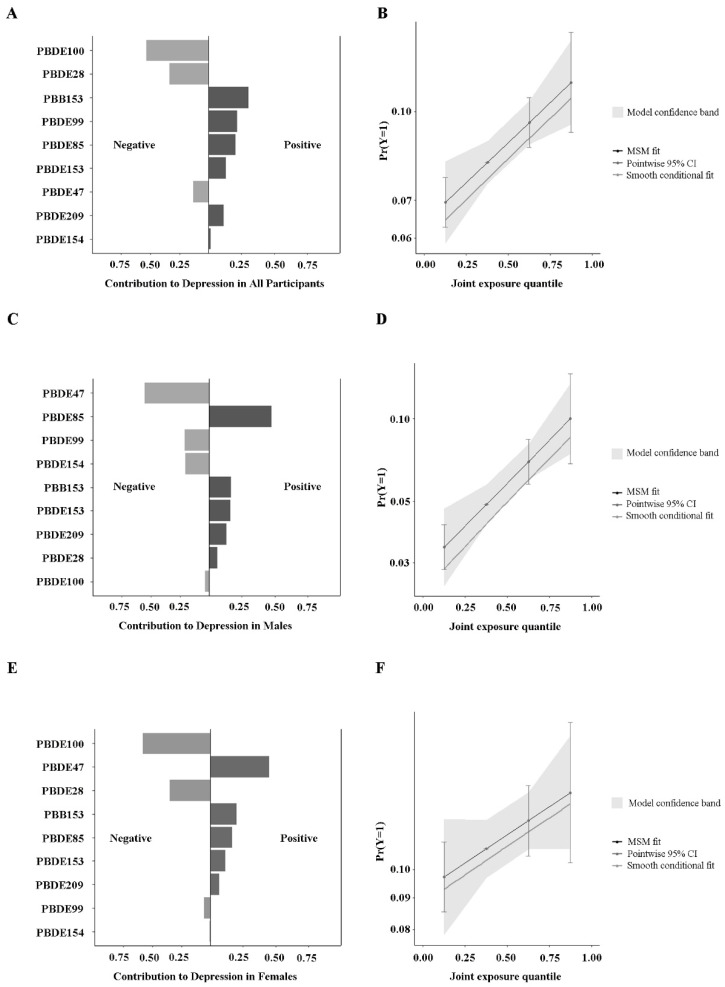
The proportion of positive and negative effects of BFRs on depression and the combined effects of mixed exposure to BFRs in the QGC model. (**A**) Proportion for depression in total participants. (**B**) Combined effects for depression in total participants. (**C**) Proportion for depression in males. (**D**) Combined effects for depression in males. (**E**) Proportion for depression in females. (**F**) Combined effects for depression in females. The size of the bar in the chart corresponds solely to its relative magnitude compared to the other impacts in the same direction. The depth of the bar graph corresponds to the overall effect size, with the darker side indicating the direction of the overall impact. All of the models are adjusted for demographic characteristics (age, race, educational levels, marital status, and PIR), lifestyle (BMI categories, cotinine levels, and alcohol consumption), and self-reported hypertension and diabetes conditions.

Figure 3 comprehensively shows the association of the BFR mixture with depression in males, females, and total participants. The joint effect of BFRs on depression and the potential impact factors were explored via QGC. The results can be summarized as follows. Mixed exposure to BFRs was positively associated with depression in all three different groups and was more significant in the whole population and male participants (*p* < 0.05). In detail, for total participants, the QGC showed a 1.176-fold ascent in depression for every one-tenth increase in the concentration of exposure to BFR mixtures (marginal OR: 1.176, 95% CI: 1.054–1.313, *p* = 0.004). In males, the QGC revealed a 1.429-fold aggrandize in the hazard of depression for every one-tenth rise in the concentration of exposure to a mixture of BFRs (marginal OR: 1.429, 95% CI: 1.165–1.754, *p* < 0.001). For female participants, the QGC displayed a 1.136-fold augment in depression for every one-tenth increase in the concentration of exposure to BFR mixtures (marginal OR: 1.136, 95% CI: 0.999–1.291, *p* = 0.050). Taken together, PBB153 and PBDE85 showed the highest importance for depression among the three groups and were potential risk factors worthy of attention.

### 3.4. Sensitivity Analyses

**Figure 4:**
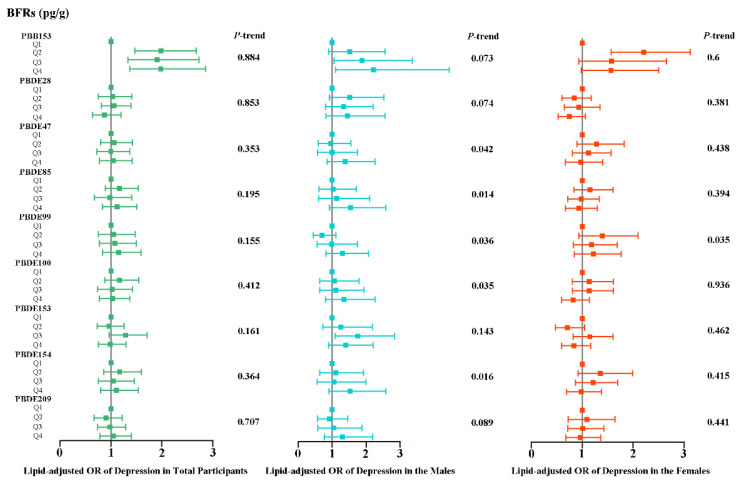
Weighted odds ratios (95% CIs) of depression by quartiles of ln-transformed serum lipid-adjusted BFR levels in all participants. The squares and horizontal lines represent the ORs and 95% CIs, respectively. All of the models are adjusted for demographic characteristics (age, race, educational levels, marital status, and PIR), lifestyle (BMI categories, cotinine levels, and alcohol consumption), and self-reported hypertension and diabetes conditions.

We reperformed all analyses using lipid-adjusted serum BFR concentrations. Figure 4 shows the weighted odds ratios (95% CIs) of depression by quartiles of ln-transformed serum lipid-adjusted BFR levels in all participants. We found that compared to PBB153, lipid-modulated PBB153 had an enhanced association with depression risk in men and an attenuated link in women. Figures S5–S7 displayed the continuous relationship of nine ln-transformed serum lipid-adjusted BFR levels associated with depression risk based on RCS analysis. The results showed that BFRs before and after lipid modulation were largely consistent with a nonlinear relationship with depression risk. Figure S8 shows the results of rating the significance of all BFRs to outcomes in the WQS model. A, B, and C are the serum BFRs before lipid adjustment, while D, E, and F are the serum BFRs after lipid adjustment, with negligible changes between the two. On the whole, PBB153 had a significant influence on the risk of depression in both men and women (*p* < 0.05), whereas PBDE85 had the greatest effect on the risk of depression in males. Table S5 shows the detailed results of the WQS model, in which mixed exposure to BFRs is significantly positively correlated with the risk of depression in the male population (OR (95%CI) was 2.185 (1.074, 4.447), *p* = 0.031). These findings are broadly compatible with those from the QGC model. Figure S9 revealed the ratio of positive and negative effects of lipid-adjusted BFRs on depression and the joint effects of exposure to mixed BFRs in the QGC model. Similarly, mixed exposure to lipid-adjusted BFRs was positively and significantly (*p* < 0.05) related to the risk of depression in the overall population and men but not significant in women. We speculate that after lipid modulation, the lipid differences between individuals are reduced, and the concentration of serum BFRs is closer to the true exposure level, so the association between BFRs and depression is somewhat attenuated. Even so, we did find a positive relationship between serum BFRs and the risk of depression in different groups.

## 4. Discussion

A comprehensive analysis of data from a large sample size was conducted to investigate the potential association between exposure to BFRs and depression among the general adult population in the United States. Specifically, the binary logistic regression and RCS revealed that, even after adjusting for various confounding variables, certain BFRs (particularly PBB153) were still significantly and positively linked to depression prevalence (*p* < 0.05). In addition, mixed exposure to BFRs was also associated with depression in the population, and this association was more significant in men (*p* < 0.05). PBB153 and PBDE85 were identified as the two BFRs with the greatest impact on outcome in the two mixed exposure models and are potential risk factors of concern.

It has long been widely accepted that there is a significant association between pollution exposure, such as exposure to airborne particulate matter, and the risk of depression. Several studies have shown that exposure to environmental mixed pollutants such as heavy metals and polycyclic aromatic hydrocarbons is positively associated with a high risk of depression [45,46,47]. Recently, a cohort study conducted in Korea found that exposure to endocrine-disrupting chemicals (EDCs) during the postpartum lactation period increased the risk of postpartum depression (PPD) in mothers and negatively affected the neurodevelopment of the fetus [48]. Animal studies have found long-term effects on the development of future mood disorders, such as anxiety and depression, resulting from prenatal exposure to nicotine and cocaine [49]. Additional in vivo experiments also found a significant association between perinatal bisphenol A (BPA) exposure and anxiety and depression [50]. On the one hand, a 28-day exposure of mice to conventional BFRs such as decabromodiphenyl ether (BDE-209), tetrabromobisphenol A (TBBPA), and hexabromocyclododecane (HBCD) revealed an upregulation of genes associated with apoptosis (caspase-3, bax, and bcl-2), a decrease in memory-related proteins (BDNF and PSD-95), and disruption of neurotransmitter pathways [51]. On the other hand, novel brominated flame retardants (NBFRs), such as Tris-(2,3-dibromo propyl) isocyanurate (TDBP-TAZTO), have been demonstrated to induce oxidative stress and concentration-dependent apoptosis in human neuroblastoma cells (SH-SY5Y cells) and to cause cognitive impairment and depressive behavior in mice in vivo and in vitro tests, respectively [52,53]. Exposure to BDE-47 in a zebrafish model has been found to lead to the development of depressive mood disorders in animal experiments with BFRs and similar substances, which to some extent supports our findings in the population [54]. Numerous studies have observed the neurotoxicity of traditional BFRs and NBFRs, which has led to a call for safer alternatives [19,55].

The potential mechanisms of environmental pollutant-induced depression, as reported in the literature, include changes in neurotransmitters, decreased brain-derived neurotrophic factor (BDNF) levels in the hippocampus, oxidative stress, neuroinflammation, and dysfunction of various functional axes [56,57,58]. Chen et al. proposed a mechanism for hypothalamic-pituitary-adrenal (HPA) axis hyperactivity in a study of depressive-like behavior in rats exposed to BPA [50]. Their work indicates persuasively that prenatal BPA exposure causes a long-term enhancement of the HPA reaction to stress, which is accompanied by evident anxiety and depression-like behaviors. In a review, Wang et al. investigated the role of microglia in the pathogenesis of depression, including how it affects the inflammatory response, neurogenesis, and regulation of tryptophan metabolism [59]. A recent study of zebrafish found that brominated flame retardant-like substances caused visual impairment by disrupting non-imaging visual (NIF) pathways, which may promote depression [54]. These ideas offer a starting point for exploring mechanisms linking BFRs and depression. Firstly, experiments on zebrafish embryos have shown that BFR analogs inhibit certain genes and proteins involved in neurodevelopment and cause neurotransmission dysfunction, ultimately reducing 5-hydroxytryptamine (5-HT) levels [60]. Alterations in monoamine neurotransmission, including serotonin, play a very important role in the onset and progression of depression [61,62]. Secondly, melatonin was found to play a protective role in neurotoxicity and apoptosis caused by BFR analogs in cellular experiments [63,64]. Melatonin is considered to be a highly cellular and neuroprotective substance that may have antidepressant functions by suppressing inflammatory responses through both immune and non-immune actions and by ameliorating the changes in brain regions associated with depression [65]. Thus, melatonin reduction due to sleep, circadian rhythm disorders, and exposure to environmental pollutants cannot be ignored in the development of depression [66,67]. Last but not least, the effect of microglia on inflammation may explain the association between BFRs and depression. In vitro experiments on BV-2 microglia from mice have shown that BFR analogs may affect microglia by influencing mitochondrial metabolism [68]. The correlation study confirmed previous findings that microglia could influence the development of depression by influencing the inflammatory response, which may be an important disease modifier that increases susceptibility to depression [59,69]. It is worth mentioning that many drugs can also exert antidepressant effects in the human body by affecting the above mechanisms, which has made a lot of contributions to the development of the medical community to fight against depression [70,71,72].

In our environmental epidemiological study, we used linear regression modeling to establish a straightforward relationship between exposure and outcome without taking into account mixed exposures to chemicals. Therefore, we introduced the QGC model to initially explore the association between mixtures as a whole and outcome. The QGC model has been employed in a multitude of ecological epidemiological studies to ascertain the impact of individual exposures on the risk posed by a combination of such exposures and has the distinct advantage of synthesizing the simplicity of inference derived from WQS regressions with the flexibility inherent in g calculations [40,73]. Therefore, both models have their strengths and can complement each other to produce richer and more reliable results.

The study was stratified by gender, and the descriptive results demonstrated that the prevalence of depression was approximately twice as high in women as in men within the study population, which is consistent with the established differences in the prevalence of depression between men and women in the United States. At the same time, logistic regression and RCS analyses showed that single BFR, especially PBB153, was more significantly associated with depression in women (*p* < 0.05). However, the results of the WQS and QGC mentioned earlier showed a higher association between exposure to mixed BFRs and depression in men than in women, which was also evident in the lipid-adjusted data. This could be linked to the anti-inflammatory and neuroprotective effects of estrogen and progesterone, which may protect women from experiencing less damage when exposed to mixed BFRs [74]. Higher serum BFR levels in men also suggested that there may be higher exposure in this group, which may have different effects on the nervous system than in women. Our findings support Rachel’s call for greater attention and research into issues related to depressive symptoms, pain, and nerve damage in men [41].

According to the official NHANES guidelines, it is recommended to use lipid-adjusted concentration data when analyzing organohalogen compounds. Therefore, we conducted a sensitivity analysis to examine the relationship between lipid-adjusted BFR data and depression outcomes. The use of lipid-moderated data weakens some important correlations, which is consistent with earlier findings on POPs [75]. However, it is necessary to note the role of lipid concentrations in these correlations due to the ease of accumulation of BFRs in lipids and the potential relevance of dyslipidemia to the pathogenesis of depression.

The study’s principal strengths are the utilization of data from the NHANES database, the study’s large sample size, the standardization of data collection, and the reliability of the information obtained. There are some limitations to our study. Firstly, this is a cross-sectional study, which means that causality cannot be established. Future prospective studies are needed to validate our findings. Secondly, although PHQ-9 is the most commonly used screening tool for depression in primary care, it is still not a complete substitute for the outcome of a clinical psychiatric specialty diagnosis. Meanwhile, it is inevitable that some degree of selection bias will be present in the data collected by NHANES, given that serum BFR samples are not collected from every individual in each cycle. Furthermore, the potential for complex interactions between the various types of BFRs, including antagonistic and synergistic effects, means that our models may not perfectly replicate the real situation. Lastly, although we considered demographic and disease variables, there may be unknown confounding variables, such as other environmental pollutants, that could affect the association between BFRs and depression.

## 5. Conclusions

In summary, our study discovered that higher concentrations of BFRs were positively linked with the risk of depression, whether exposed to a single BFR or a mixture of BFRs, with PBB153 and PBDE85 being key contributors. Although these results suggest that reducing BFR exposure may be a way to mitigate the risk of depression in the population, further research is required in the form of large-scale prospective studies to gain a deeper understanding of the effects and mechanisms of BFRs on depression in the population, especially in males, and to provide reliable evidence for the development of policies related to mental health problems caused by BFRs exposure. Finally, in clinical practice, it is necessary to strengthen the monitoring of high-risk groups with the potential risk of depression and focus on individuals with a history of BFR exposure. Meanwhile, in daily life, this study recommends strengthening individual awareness of BFR toxicity and the rational use of BFRS-doped products, as well as minimizing contact with BFRS-rich household dust (such as regular cleaning of the house, dust removal, increased outdoor activity time, etc.).

## Figures and Tables

**Table 1:** Baseline characteristics of the study population, NHANES 2005–2016 (N = 8138).

Catalogs	Total Population			Male			Female		
	Depression			Depression			Depression		
	No	Yes	*p*-Value	No	Yes	*p*-Value	No	Yes	*p*-Value
**Number of subjects (%) ^a^**	7422 (91.2)	716 (8.8)		3736 (94.0)	239 (6.0)		3686 (88.5)	477 (11.5)	
**Age (%) ^a^**			0.028 *			0.262			0.073
20–40 years	2590 (34.9)	242 (33.8)		1328 (35.5)	76 (31.8)		1262 (34.2)	166 (34.8)	
40–60 years	2390 (32.2)	264 (36.9)		1177 (31.5)	87 (36.4)		1213 (32.9)	177 (37.1)	
≥60 years	2442 (32.9)	210 (29.3)		1231 (32.9)	76 (31.8)		1211 (32.9)	134 (28.1)	
**Race (%) ^a^**			<0.001 *			0.617			<0.001 *
Mexican American	1157 (15.6)	114 (15.9)		587 (15.7)	39 (16.3)		570 (15.5)	75 (15.7)	
Other Hispanic	675 (9.1)	101 (14.1)		331 (8.9)	27 (11.3)		344 (9.3)	74 (15.5)	
Non-Hispanic White	3353 (45.2)	299 (41.8)		1690 (45.2)	100 (41.8)		1663 (45.1)	199 (41.7)	
Non-Hispanic Black	1542 (20.8)	156 (21.8)		773 (20.7)	53 (22.2)		769 (20.9)	103 (21.6)	
Other race	695 (9.4)	46 (6.4)		355 (9.5)	20 (8.4)		340 (9.2)	26 (5.5)	
**Educational level (%) ^a^**			<0.001 *			<0.001 *			<0.001 *
Below high school	1714 (23.1)	247 (34.5)		916 (24.5)	87 (36.4)		798 (21.6)	160 (33.5)	
High school	1684 (22.7)	172 (24.0)		893 (23.9)	62 (25.9)		791 (21.5)	110 (23.1)	
Above high school	4024 (54.2)	297 (41.5)		1927 (51.6)	90 (37.7)		2097 (56.9)	207 (43.4)	
**Marital status (%) ^a^**			<0.001 *			<0.001 *			<0.001 *
Married/living with partner	4591 (61.9)	334 (46.6)		2486 (66.5)	128 (53.6)		2105 (57.1)	206 (43.2)	
Widowed/divorced/separated/never married	2831 (38.1)	382 (53.4)		1250 (33.5)	111 (46.4)		1581 (42.9)	271 (56.8)	
**Poverty income ratio (%) ^a^**			<0.001 *			<0.001 *			<0.001 *
≤1.3	2127 (28.7)	379 (52.9)		1030 (27.6)	123 (51.5)		1097 (29.8)	256 (53.7)	
1.3–3.5	2820 (38.0)	238 (33.2)		1424 (38.1)	79 (33.1)		1396 (37.9)	159 (33.3)	
>3.5	2475 (33.3)	99 (13.8)		1282 (34.3)	37 (15.5)		1193 (32.4)	62 (13.0)	
**Body mass index (%) ^a^**			<0.001 *			0.062			<0.001 *
<25 kg/m^2^	2155 (29.0)	168 (23.5)		992 (26.6)	61 (25.5)		1163 (31.6)	107 (22.4)	
25–30 kg/m^2^	2537 (34.2)	197 (27.5)		1479 (39.6)	80 (33.5)		1058 (28.7)	117 (24.5)	
≥30 kg/m^2^	2730 (36.8)	351 (49.0)		1265 (33.9)	98 (41.0)		1465 (39.7)	253 (53.0)	
**Cotinine level (%) ^a^**			<0.001 *			0.001 *			<0.001 *
Below LLOD	1963 (26.4)	121 (16.9)		777 (20.8)	28 (11.7)		1186 (32.2)	93 (19.5)	
Above LLOD	5459 (73.6)	595 (83.1)		2959 (79.2)	211 (88.3)		2500 (67.8)	384 (80.5)	
**Alcohol consumption (%) ^a^**			0.591			0.419 *			0.174
12 drinks or fewer	5352 (72.1)	509 (71.1)		622 (16.6)	35 (14.6)		1448 (39.3)	172 (36.1)	
More than 12 drinks	2070 (27.9)	207 (28.9)		3114 (83.4)	204 (85.4)		2238 (60.7)	305 (63.9)	
**Hypertension (%) ^a^**	2594 (35.0)	322 (45.0)	<0.001 *	1300 (34.8)	105 (43.9)	0.004 *	1294 (35.1)	217 (45.5)	<0.001 *
**Diabetes (%) ^a^**	862 (11.6)	144 (20.1)	<0.001 *	456 (12.2)	41 (17.2)	0.025 *	406 (11.0)	103 (21.6)	<0.001 *

^a^ Number of participants and percentage. Chi-square test was used to compare the differences in categorical variables between participants with and without Depression. * indicates that after the Benjamini-Hochberg false discovery rate (FDR) correction, the *p*-trend < 0.05.

**Table 2:** Geometric means and quartiles of serum BFRs. NHANES 2005–2016 (N = 8138).

Serum BFRs (pg/g)	GM (95% CI) ^a^	Median (IQR) ^b^
PBB153	15.907 (15.534, 16.297)	16.980 (7.941, 29.870)
PBDE28	7.410 (7.308, 7.516)	7.362 (4.854, 11.200)
PBDE47	133.188 (131.236, 135.233)	126.900 (83.860, 205.300)
PBDE85	2.753 (2.708, 2.798)	2.549 (1.573, 4.369)
PBDE99	26.457 (25.997, 26.934)	24.890(15.160, 41.840)
PBDE100	27.321 (26.897, 27.743)	25.500 (16.480, 41700)
PBDE153	55.855 (55.037, 56.713)	52.260 (34.300, 87.070)
PBDE154	2.530 (2.489, 2.573)	2.342 (1.580, 4.005)
PBDE209	16.301 (16.119, 16.478)	17.600 (12.030, 17.940)

Note: PBB153: 2,2′,4,4′,5,5′-Hexabromobiphenyl; PBDE28: 2,4,4′-Tribromodiphenyl ether; PBDE47: 2,2′,4,4′-Tetrabromodiphenyl ether; PBDE85: 2,2′,3,4,4′-Tentabromodiphenyl ether; PBDE99: 2,2′,4,4′,5-Pentabromodiphenyl ether; PBDE100: 2,2′,4,4′,6-Pentabromodiphenyl ether; PBDE153: 2,2′,4,4′,5,5′-Hexabromodiphenyl ether; PBDE154: 2,2′,4,4′,5,6′-Hexabromodiphenyl ether; PBDE209: Decabromodiphenyl ether. ^a^ G-Mean (95%). ^b^ Median (25th, 75th percentiles).

## Data Availability

Data is publicly available on the NHANES website.

## Supplementary Materials

The following supporting information can be downloaded at: https://www.mdpi.com/article/10.3390/toxics12120918/s1, Table S1. The differences between participants with and without complete BFRs data. NHANES 2005-2016; Table S2. The concentrations of serum BFRs in Male and Female subgroups. NHANES 2005-2016; Table S3. Detection rates of serum BFRs. NHANES 2005-2016 (N = 8138); Table S4. Geometric means and geometric standard deviations of serum BFRs by each cycle of NHANES (2005-2016); Table S5. Associations of BFRs index with depression by WQS regression, NHANES 2005-2016 (N = 8138); Figure S1. Pearson correlation coefficients between serum BFRs; Figure S2. The continuous relationship of nine ln-transformed serum BFRs levels associated with depression risk in all participants based on RCS analysis; Figure S3. The continuous relationship of nine ln-transformed serum BFRs levels associated with depression risk in males based on RCS analysis; Figure S4. The continuous relationship of nine ln-transformed serum BFRs levels associated with depression risk in females based on RCS analysis; Figure S5. The continuous relationship of nine ln-transformed serum lipid-adjusted BFRs levels associated with depression risk in all participants based on RCS analysis; Figure S6. The continuous relationship of nine ln-transformed serum lipid-adjusted BFRs levels associated with depression risk in males based on RCS analysis; Figure S7. The continuous relationship of nine ln-transformed serum lipid-adjusted BFRs levels associated with depression risk in females based on RCS analysis; Figure S8. Association of BFRs mixture in WQS regression (positive direction) with depression risk; Figure S9. The proportion of positive and negative effects of lipid-adjusted BFRs on depression and the combined effects of mixed exposure to BFRs in the QGC model.
