# Supplementary material for: Associations Between Brominated Flame Retardant Exposure and Depression in Adults: A Cross-Sectional Study
Source: Toxics. 2024 Dec 19;12(12):918. doi: 10.3390/toxics12120918 (PMC11728815; doi:10.3390/toxics12120918)
Supplement: Supplementary file 1 [file toxics-12-00918-s001.zip › toxics-3379462-supplementary.pdf]

Lists of Table

Table S1. The differences between participants with and without complete BFRs data. NHANES 2005-2016.

Table S2. The concentrations of serum BFRs in Male and Female subgroups. NHANES 2005-2016.

Table S3. Detection rates of serum BFRs. NHANES 2005-2016 (N = 8138).

Table S4. Geometric means and geometric standard deviations of serum BFRs by each cycle of NHANES (2005-2016).

Table S5. Associations of BFRs index with depression by WQS regression, NHANES 2005-2016 (N = 8138).

Lists of Figure

Figure S1. Pearson correlation coefficients between serum BFRs.

Figure S2. The continuous relationship of nine ln-transformed serum BFRs levels associated with depression risk in all participants based on RCS analysis.

Figure S3. The continuous relationship of nine ln-transformed serum BFRs levels associated with depression risk in males based on RCS analysis.

Figure S4. The continuous relationship of nine ln-transformed serum BFRs levels associated with depression risk in females based on RCS analysis.

Figure S5. The continuous relationship of nine ln-transformed serum lipid-adjusted BFRs levels associated with depression risk in all participants based on RCS analysis.

Figure S6. The continuous relationship of nine ln-transformed serum lipid-adjusted

BFRs levels associated with depression risk in males based on RCS analysis.

Figure S7. The continuous relationship of nine ln-transformed serum lipid-adjusted BFRs levels associated with depression risk in females based on RCS analysis.

Figure S8. Association of BFRs mixture in WQS regression (positive direction) with depression risk.

Figure S9. The proportion of positive and negative effects of lipid-adjusted BFRs on depression and the combined effects of mixed exposure to BFRs in the QGC model.

Table S1. The differences between participants with and without complete BFRs data. NHANES 2005-2016.

Catalogs	Two different population samples		*P*-value
	With complete BFRs data	Without complete BFRs data	
**Number of subjects (%)** [a]	8138 (31.9)	17397 (68.1)	
**Gender (%)** [a]			0.507
Male	3975 (48.8)	8575 (49.3)	
Female	4163 (51.2)	8822 (50.7)	
**Age (%)** [a]			0.618
20-40 years	2832 (34.8)	5991 (34.4)	
40-60 years	2654 (32.6)	5781 (33.2)	
≥60 years	2652 (32.6)	5625 (32.3)	
**Race (%)** [a]			0.146
Mexican American	1271 (15.6)	2734 (15.7)	
Other Hispanic	776 (9.5)	1552 (8.9)	
Non-Hispanic White	3652 (44.9)	8054 (46.3)	
Non-Hispanic Black	1698 (20.9)	3480 (20.0)	
Other race	741 (9.1)	1577 (9.1)	
**Educational level (%)** [a]			0.973
Below high school	1961 (24.1)	4172 (24.0)	
High school	1856 (22.8)	3985 (22.9)	
Above high school	4321 (53.1)	9240 (53.1)	
**Marital status (%)** [a]			0.934
Married/living with partner	4925 (60.5)	10519 (60.5)	
Widowed/divorced/separated/never married	3213 (39.5)	6878 (39.5)	
**Poverty income ratio (%)** [a]			0.385
≤1.3	2506 (30.8)	5499 (31.6)	
1.3–3.5	3058 (37.6)	6503 (37.4)	
>3.5	2574 (31.6)	5395 (31.0)	
**Body mass index (%)** [a]			0.840
<25 kg/m^2	2323 (28.5)	5000 (28.7)	
25–30 kg/m^2	2734 (33.6)	5780 (33.2)	
≥30 kg/m^2	3081 (37.9)	6617 (38.0)	
**Cotinine level (%)** [a]			0.503
Below LLOD	2084 (25.6)	4387 (25.2)	
Above LLOD	6054 (74.4)	13010 (74.8)	
**Alcohol consumption (%)** [a]			0.982
12 drinks or fewer	2277 (28.0)	4870 (28.0)	
More than 12 drinks	5861 (72.0)	12527 (72.0)	

**Hypertension (%)** [a]	2916 (35.8)	6136 (35.3)	0.382
**Diabetes (%)** [a]	1006 (12.4)	2116 (12.2)	0.651
**Depression (%)** [a]	716 (8.8)	1498 (8.6)	0.620

a Number of participants and percentage. Chi-square test was used to compare the differences of categorical variables between participants with and without complete BFRs data.

Table S2. The concentrations of serum BFRs in Male and Female subgroups. NHANES 2005-2016.

	Male	Female	
BFRs (pg/g)	GM (95%CI)	GM (95%CI)	*p*
PBB153	20.001<br>(19.327, 20.700)	12.782<br>(12.379, 13.198)	< 0.001
PBDE28	7.707<br>(7.558, 7.859)	7.138<br>(7.001, 7.277)	< 0.001
PBDE47	143.636<br>(140.602, 146.736)	123.922<br>(121.337, 126.562)	< 0.001
PBDE85	2.951<br>(2.882, 3.021)	2.576<br>(2.518, 2.636)	< 0.001
PBDE99	29.094<br>(28.379, 29.826)	24.162<br>(23.583, 24.756)	< 0.001
PBDE100	29.356<br>(28.722, 30.005)	25.510<br>(24.983, 26.048)	< 0.001
PBDE153	66.094<br>(64.729, 67.488)	47.563<br>(46.604, 48.542)	< 0.001
PBDE154	2.747<br>(2.683, 2.812)	2.340<br>(2.288, 2.393)	< 0.001
PBDE209	18.049<br>(17.759, 18.343)	14.790<br>(14.569, 15.013)	< 0.001

Note： PBB153: 2,2´,4,4´,5,5´-Hexabromobiphenyl; PBDE28: 2,4,4´-Tribromodiphenyl ether; PBDE47: 2,2´,4,4´-Tetrabromodiphenyl ether; PBDE85: 2,2´,3,4,4´-Tentabromodiphenyl ether; PBDE99: 2,2´,4,4´,5-Pentabromodiphenyl ether; PBDE100: 2,2´,4,4´,6-Pentabromodiphenyl ether; PBDE153: 2,2´,4,4´,5,5´-Hexabromodiphenyl ether; PBDE154: 2,2´,4,4´,5,6´-Hexabromodiphenyl ether; PBDE209: Decabromodiphenyl ether. The Student-t test was used to compare the difference in mean serum BFRs between different sex groups.

Table S3. Detection rates of serum BFRs. NHANES 2005-2016 (N = 8138).

Serum BFRs (pg/g)	**2005-2006**			**2007-2008**			**2009-2010**		
	Pool number	<LLOD	Detection rate (%)	Pool number	<LLOD	Detection rate (%)	Pool number	<LLOD	Detection rate (%)
PBB153	247	33	86.64	264	19	92.80	301	39	87.04
PBDE28	247	3	98.79	264	0	100.00	301	7	97.67
PBDE47	247	0	100.00	264	0	100.00	301	0	100.00
PBDE85	247	43	82.59	264	58	78.03	301	34	88.70
PBDE99	247	0	100.00	264	0	100.00	301	0	100.00
PBDE100	247	0	100.00	264	0	100.00	301	0	100.00
PBDE153	247	0	100.00	264	0	100.00	301	0	100.00
PBDE154	247	46	81.38	264	72	72.73	301	61	79.73
PBDE209	247	217	12.15	264	236	10.61	301	12	96.01
	**2011-2012**			**2013-2014**			**2015-2016**		
	Pool number	<LLOD	Detection rate (%)	Pool number	<LLOD	Detection rate (%)	Pool number	<LLOD	Detection rate (%)
PBB153	251	15	94.02	284	22	92.25	265	45	83.02
PBDE28	251	11	95.62	284	0	100.00	265	34	87.17
PBDE47	251	0	100.00	284	0	100.00	265	0	100.00
PBDE85	251	29	88.45	284	35	87.68	265	168	36.30
PBDE99	251	0	100.00	284	00	100.00	265	0	100.00
PBDE100	251	0	100.00	284	0	100.00	265	0	100.00
PBDE153	251	0	100.00	284	0	100.00	265	0	100.00
PBDE154	251	25	90.04	284	37	86.97	265	120	54.72
PBDE209	251	4	98.41	284	6	97.89	265	10	96.23

Note：PBB153: 2,2´,4,4´,5,5´-Hexabromobiphenyl; PBDE28: 2,4,4´-Tribromodiphenyl ether; PBDE47: 2,2´,4,4´-Tetrabromodiphenyl ether;

PBDE85: 2,2´,3,4,4´-Tentabromodiphenyl ether; PBDE99: 2,2´,4,4´,5-Pentabromodiphenyl ether; PBDE100: 2,2´,4,4´,6-Pentabromodiphenyl ether; PBDE153: 2,2´,4,4´,5,5´-Hexabromodiphenyl ether; PBDE154: 2,2´,4,4´,5,6´-Hexabromodiphenyl ether; PBDE209: Decabromodiphenyl ether. LLOD: lower limit of detection.

Table S4. Geometric means and geometric standard deviations of serum BFRs by each cycle of NHANES (2005-2016).

Serum BFRs (pg/g)	Cycles of NHANES												*P* for trend
	2005-2006 n = 1973		2007-2008 n = 2070		2009-2010 n = 2290		2011-2012 n = 1910		2013-2014 n = 2141		2015-2016 n = 1989		
	Mean	standard deviation	Mean	standard deviation	Mean	standard deviation	Mean	standard deviation	Mean	standard deviation	Mean	standard deviation	
PBB153	34.439	77.980	32.634	62.765	23.060	35.875	19.517	30.112	25.656	67.512	15.450	39.141	0.060
PBDE28	12.442	7.971	11.447	7.691	8.554	4.997	7.219	4.457	7.215	4.814	5.128	4.241	0.008[a]
PBDE47	291.364	264.032	212.412	156.780	173.538	129.830	146.460	104.282	128.053	100.187	104.101	96.277	0.008[a]
PBDE85	6.708	7.891	5.097	4.447	3.463	3.424	2.947	2.682	2.821	2.681	2.474	2.648	0.008[a]
PBDE99	71.043	78.690	47.494	43.026	37.491	43.901	32.226	31.441	26.849	27.940	23.180	30.910	0.008[a]
PBDE100	57.798	56.836	44.469	35.171	35.264	26.428	29.684	22.144	25.972	18.073	22.930	22.212	0.008[a]
PBDE153	73.261	62.156	75.326	59.860	71.663	63.530	65.889	69.458	62.437	46.713	64.289	50.727	0.060
PBDE154	6.188	6.333	4.444	3.538	3.301	3.198	2.861	2.585	2.449	2.145	2.082	2.323	0.008[a]
PBDE209	21.389	14.615	20.041	10.466	24.110	57.430	19.298	15.791	14.741	9.918	13.894	13.330	0.060

[a] :P＜0.05 in the Mann-Kendall test.

Table S5. Associations of BFRs index with depression by WQS regression, NHANES 2005-2016 (N = 8138).

	Direction	WQS model regression index weights									OR (95%CI)	P-value
Serum BFRs												
Total participants	positive	0.436	0.158	0.135	0.096	0.074	0.063	0.018	0.016	0.004	0.965 (0.653,1.426)	0.858
	negative	0.492	0.174	0.104	0.099	0.066	0.036	0.013	0.010	0.006	1.062 (0.730,1.543)	0.754
Males	positive	0.379	0.192	0.149	0.133	0.100	0.028	0.018	0.001	0.000	**2.185 (1.074,4.447)**	**0.031**
	negative	0.413	0.306	0.079	0.077	0.057	0.032	0.027	0.009	0.000	1.306 (0.727,2.346)	0.372
Females	positive	0.412	0.300	0.165	0.032	0.026	0.024	0.019	0.018	0.004	0.821 (0.513,1.314)	0.411
	negative	0.293	0.217	0.143	0.123	0.085	0.067	0.065	0.006	0.001	1.347 (0.897,2.023)	0.151
Lipid-adjusted serum BFRs												
Total participants	positive	0.277	0.230	0.222	0.106	0.088	0.026	0.025	0.014	0.011	1.134 (0.760,1.692)	0.537
	negative	0.449	0.238	0.113	0.104	0.065	0.014	0.010	0.007	0.000	0.898 (0.609,1.323)	0.586
Males	positive	0.418	0.280	0.153	0.114	0.016	0.010	0.008	0.001	0.000	1.708 (0.869,3.359)	0.121
	negative	0.382	0.222	0.181	0.114	0.061	0.024	0.012	0.004	0.000	1.294 (0.743,2.256)	0.360
Females	positive	0.458	0.308	0.098	0.085	0.048	0.002	0.001	0.001	0.000	1.441 (0.900,2.308)	0.128
	negative	0.348	0.213	0.169	0.099	0.077	0.070	0.015	0.008	0.000	1.226 (0.789,1.904)	0.364

Notes: WQS: weighted quantile sum; OR: odds ratio; CI: confidence interval. All of the models are adjusted for demographic characteristics (gender, age, race, educational levels, marital status and PIR), lifestyle (BMI categories, cotinine levels and alcohol consumption) and self-reported of hypertension and diabetes conditions.

Figure S1

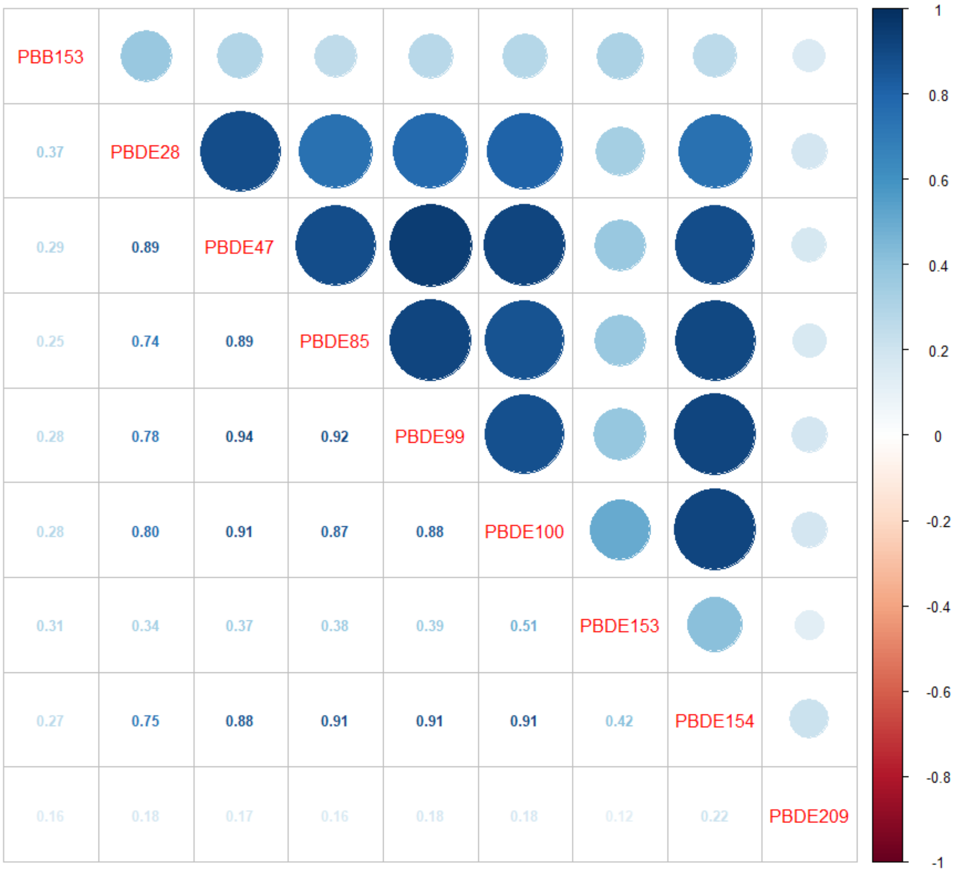

Figure S1. Pearson correlation coefficients between serum BFRs.

Figure S2

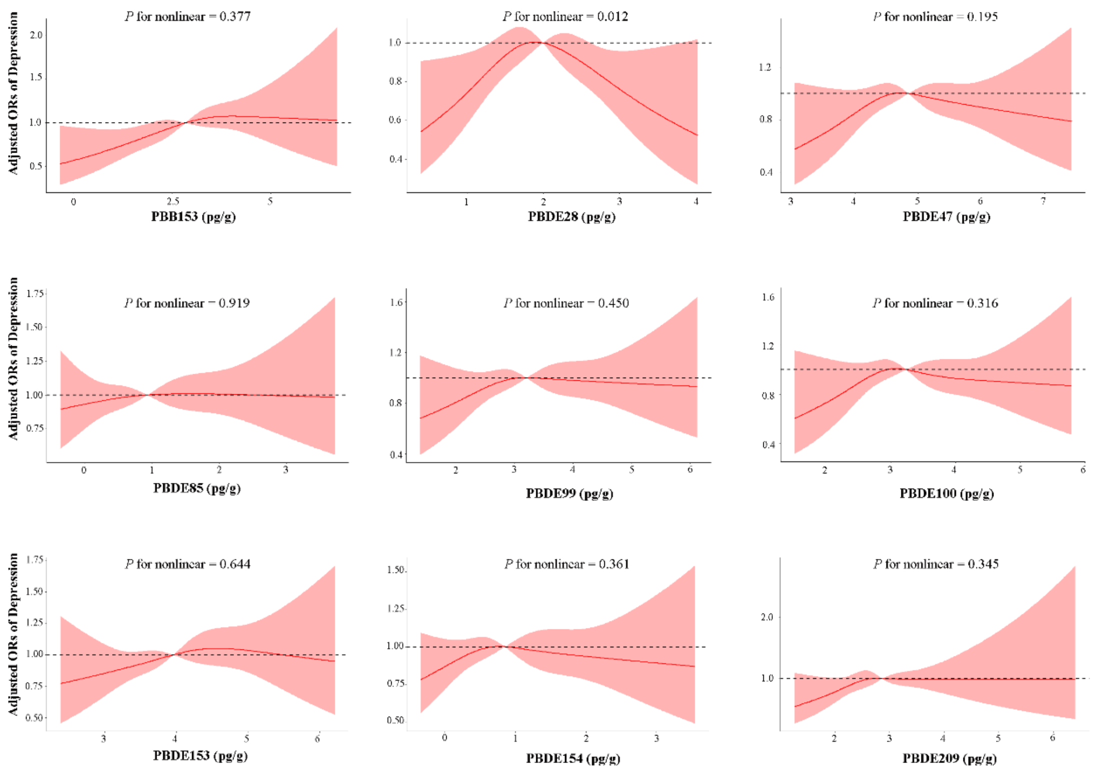

Figure S2. The continuous relationship of nine ln-transformed serum BFRs levels associated with depression risk in all participants based on RCS analysis. The red solid lines represent the ORs, and red shadow range represent the 95% CIs. The horizontal dashed line represents to the reference odds ratio of 1.0. All of the models are adjusted for demographic characteristics (gender, age, race, educational levels, marital status and PIR), lifestyle (BMI categories, cotinine levels and alcohol consumption) and self-reported of hypertension and diabetes conditions.

Figure S3

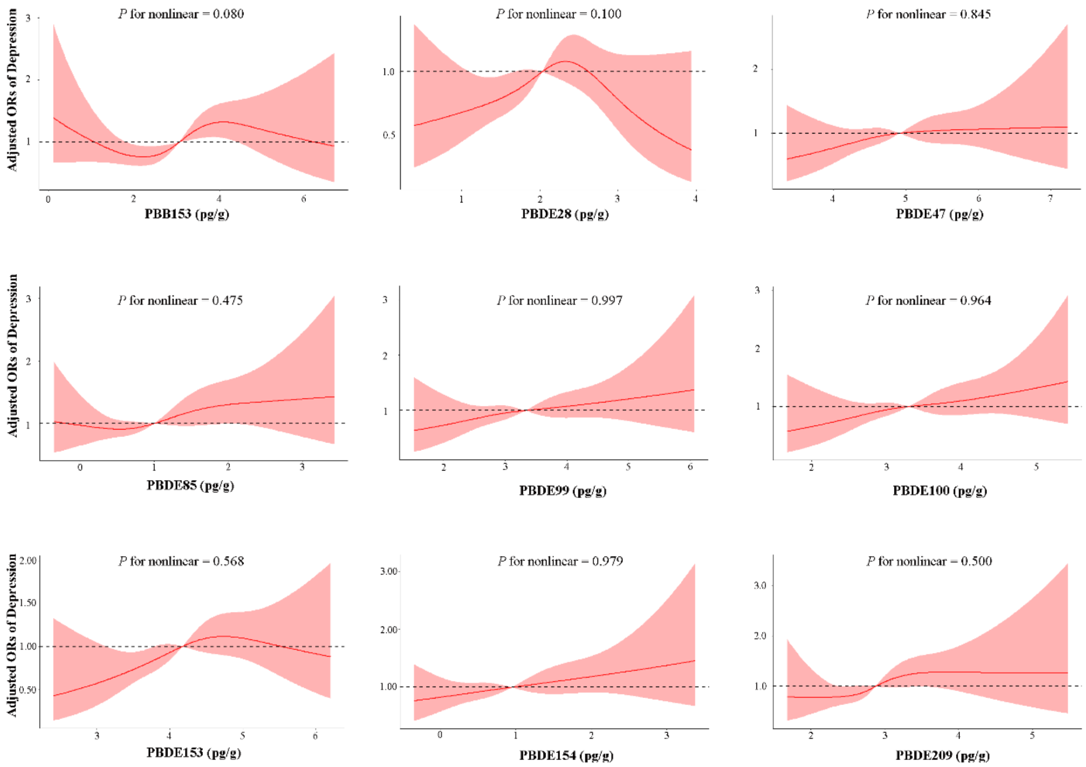

Figure S3. The continuous relationship of nine ln-transformed serum BFRs levels associated with depression risk in males based on RCS analysis. The red solid lines represent the ORs, and red shadow range represent the 95% CIs. The horizontal dashed line represents to the reference odds ratio of 1.0. All of the models are adjusted for demographic characteristics (age, race, educational levels, marital status and PIR), lifestyle (BMI categories, cotinine levels and alcohol consumption) and self-reported of hypertension and diabetes conditions.

Figure S4

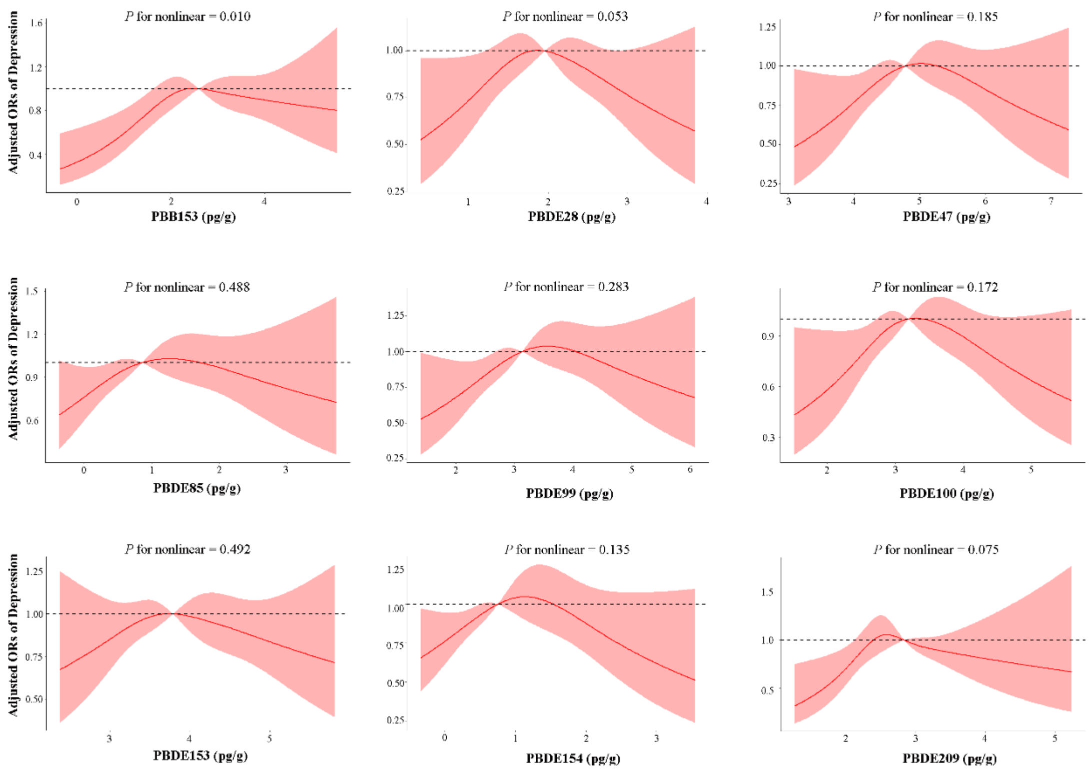

Figure S4. The continuous relationship of nine ln-transformed serum BFRs levels associated with depression risk in females based on RCS analysis. The red solid lines represent the ORs, and red shadow range represent the 95% CIs. The horizontal dashed line represents to the reference odds ratio of 1.0. All of the models are adjusted for demographic characteristics (age, race, educational levels, marital status and PIR), lifestyle (BMI categories, cotinine levels and alcohol consumption) and self-reported of hypertension and diabetes conditions.

Figure S5

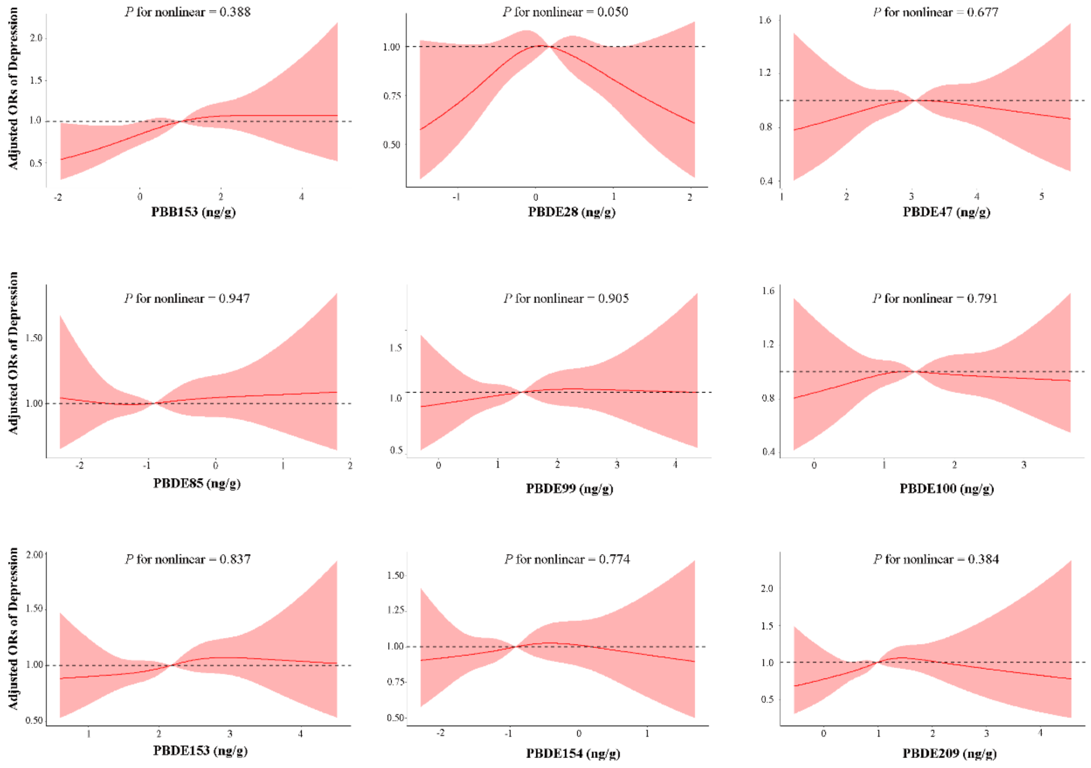

Figure S5. The continuous relationship of nine ln-transformed serum lipid-adjusted BFRs levels associated with depression risk in all participants based on RCS analysis. The red solid lines represent the ORs, and red shadow range represent the 95% CIs. The horizontal dashed line represents to the reference odds ratio of 1.0. All of the models are adjusted for demographic characteristics (gender, age, race, educational levels, marital status and PIR), lifestyle (BMI categories, cotinine levels and alcohol consumption) and self-reported of hypertension and diabetes conditions.

Figure S6

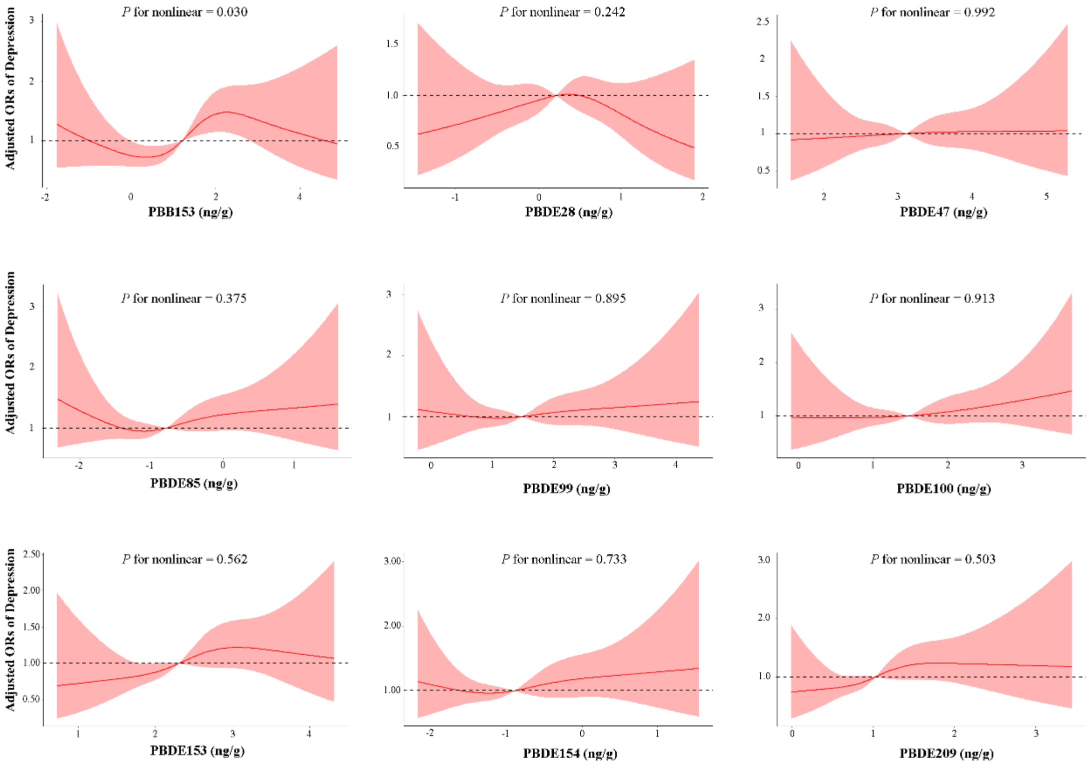

Figure S6. The continuous relationship of nine ln-transformed serum lipid-adjusted BFRs levels associated with depression risk in males based on RCS analysis. The red solid lines represent the ORs, and red shadow range represent the 95% CIs. The horizontal dashed line represents to the reference odds ratio of 1.0. All of the models are adjusted for demographic characteristics (age, race, educational levels, marital status and PIR), lifestyle (BMI categories, cotinine levels and alcohol consumption) and self-reported of hypertension and diabetes conditions.

Figure S7

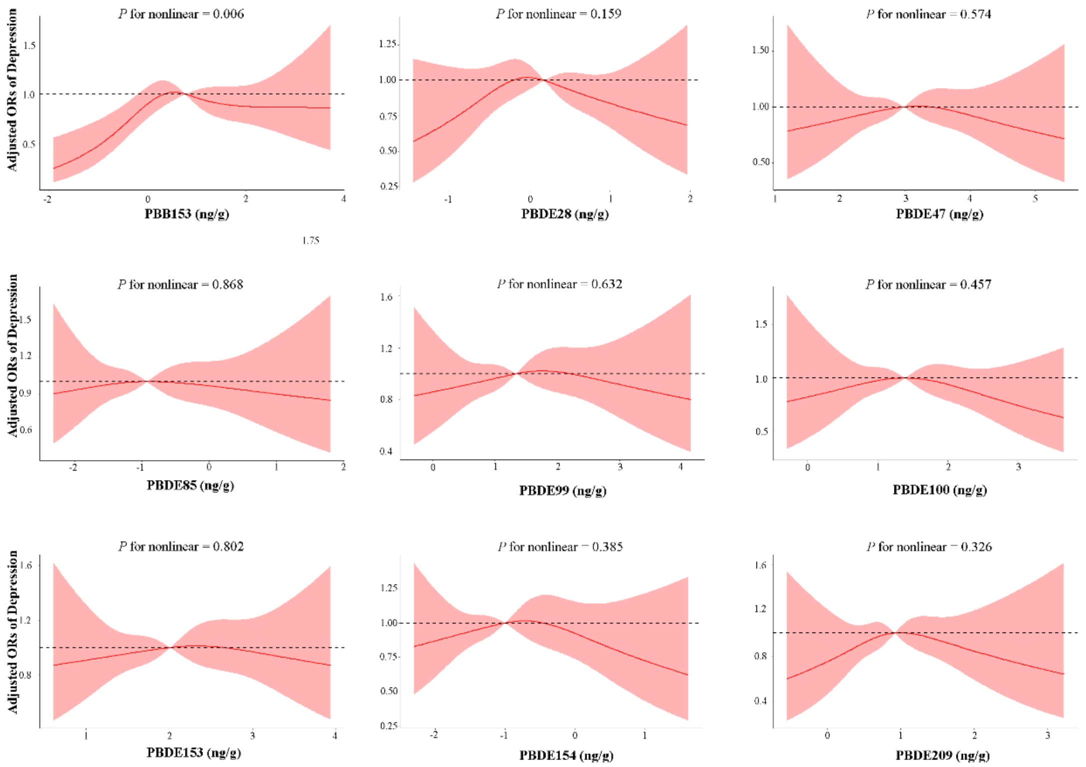

Figure S7. The continuous relationship of nine ln-transformed serum lipid-adjusted BFRs levels associated with depression risk in females based on RCS analysis. The red solid lines represent the ORs, and red shadow range represent the 95% CIs. The horizontal dashed line represents to the reference odds ratio of 1.0. All of the models are adjusted for demographic characteristics (age, race, educational levels, marital status and PIR), lifestyle (BMI categories, cotinine levels and alcohol consumption) and self-reported of hypertension and diabetes conditions.

Figure S8

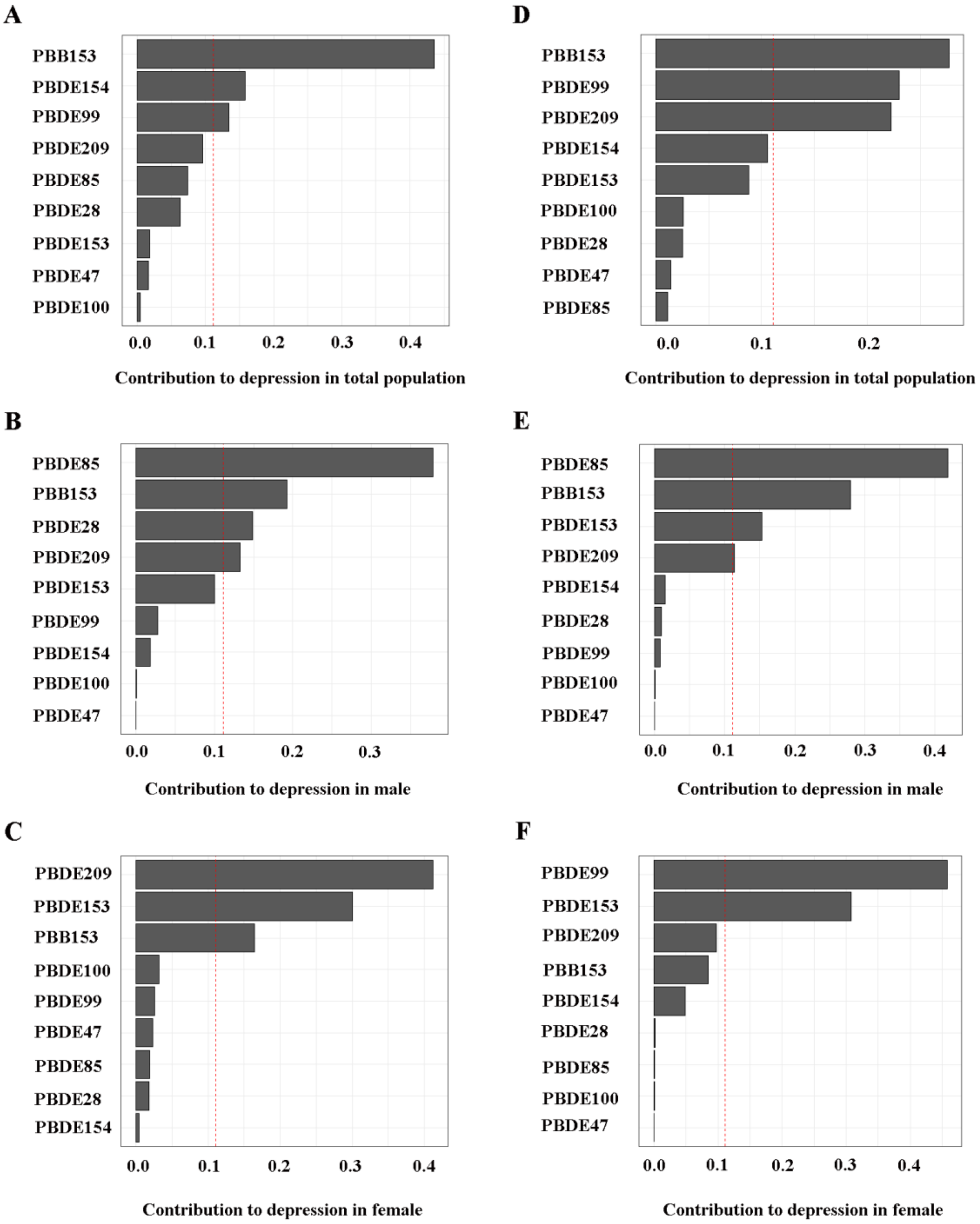

Figure S8. Association of BFRs mixture in WQS regression (positive direction) with depression risk. (D), (E) and (F) showed the relationship between **lipid-regulated** BFRs and depression. All of the models are adjusted for demographic characteristics (age, race, educational levels, marital status and PIR), lifestyle (BMI categories, cotinine levels and alcohol consumption) and self-reported of hypertension and diabetes conditions.

Figure S9

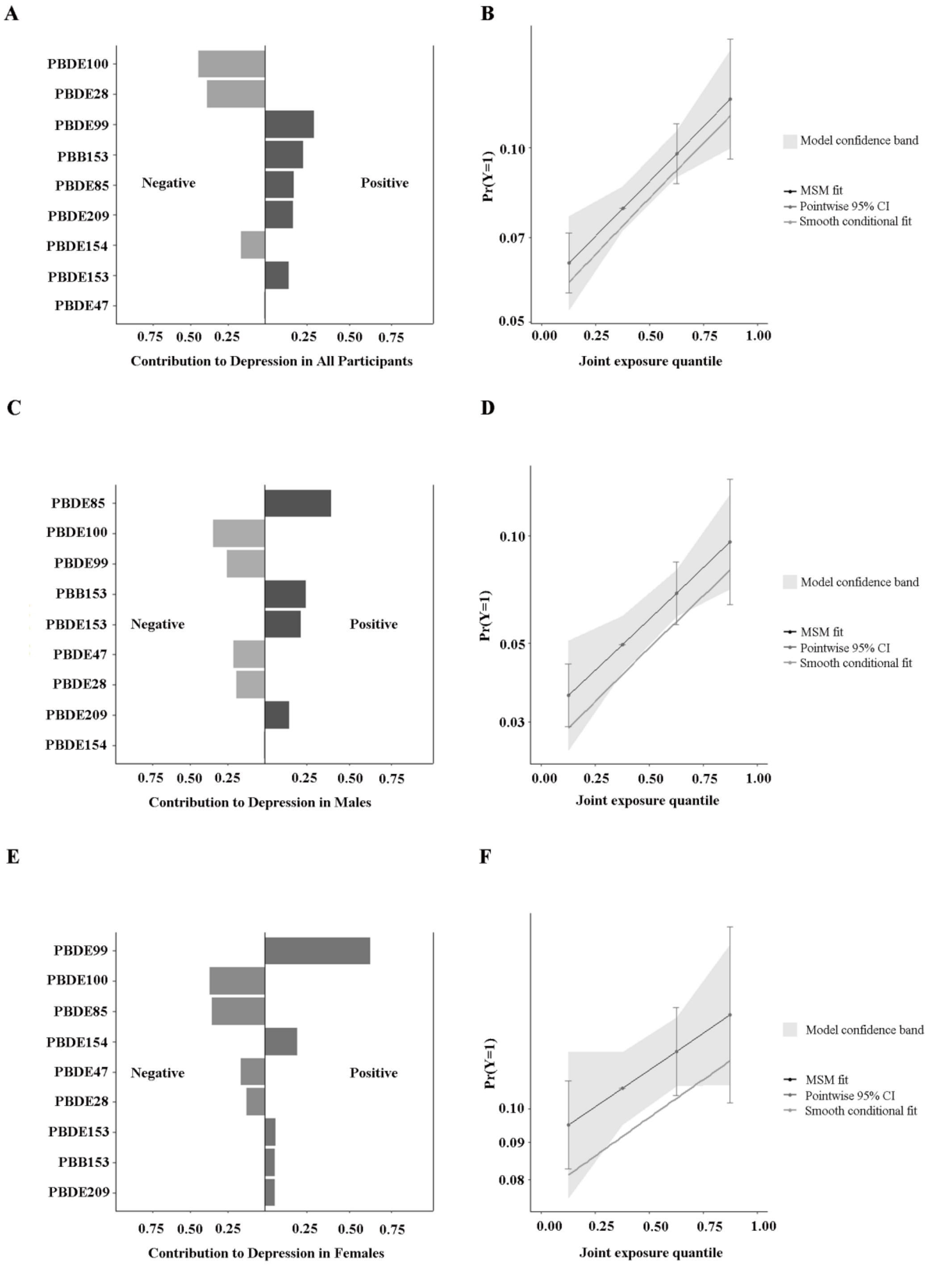

Figure S9. The proportion of positive and negative effects of lipid-adjusted BFRs on depression and the combined effects of mixed exposure to BFRs in the QGC model. (A) Proportion for depression in total participants; (B) Combined effects for depression in total participants; (C) Proportion for depression in males; (D) Combined

effects for depression in males; (E) Proportion for depression in females; (F) Combined effects for depression in females. All of the models are adjusted for demographic characteristics (age, race, educational levels, marital status and PIR), lifestyle (BMI categories, cotinine levels and alcohol consumption) and self-reported of hypertension and diabetes conditions.
